# Abdominal Cerclage in Twin Pregnancy after Radical Surgical Conization

**DOI:** 10.1155/2014/519826

**Published:** 2014-01-28

**Authors:** Ioannis Kyvernitakis, Fred Lotgering, Birgit Arabin

**Affiliations:** ^1^Department of Prenatal and Perinatal Medicine, Clara Angela Foundation, Koenigsallee 36, 14193 Berlin, Germany; ^2^Department of Pre- and Perinatal Medicine, Center of Mother and Child, University Hospital of Giessen and Marburg, Philipps University of Marburg, Campus Marburg, Baldinger Straße 1, 35043 Marburg, Germany; ^3^Department of Obstetrics and Gynecology, Radboudumc, Geert Grooteplein 10, 6525 GA Nijmegen, The Netherlands

## Abstract

Radical and repeated cone biopsies are associated with a high risk of spontaneous preterm birth. A 30-year-old gravida 1 presented with a spontaneous dichorionic twin pregnancy. She had a history of two radical surgical conizations. By speculum examination, no cervical tissue was detected. A history-indicated transabdominal cervicoisthmic cerclage was performed at 12 + 4/7 gestational weeks because of assumed cervicoisthmic insufficiency. The pregnancy continued until 34 + 3/7 weeks when the patient developed preeclampsia indicating Cesarean delivery. Transabdominal cerclage in twin pregnancy has rarely been described, but it may be considered in case of extreme cervical shortening after radical cervical surgery, as it would in singleton pregnancy.

## 1. Introduction

Benson and Durfee first described transabdominal cerclage in 1965 [1]. In 1987, Wallenburg and Lotgering [2] and later Lotgering et al. [3] discussed indications for transabdominal cervicoisthmic cerclage (TAC) and suggested its use in case of suspected cervical insufficiency in women with a very short cervix to allow effective transvaginal cerclage. This may include the congenitally short or amputated cervix.

At present, few case reports (Table 1) have described the use of TAC in twin pregnancies. We report on a case in which TAC was performed in a nulliparous patient with twin pregnancy and a history of two radical surgical conizations.

## 2. Presentation of the Case

**Figure 1:**
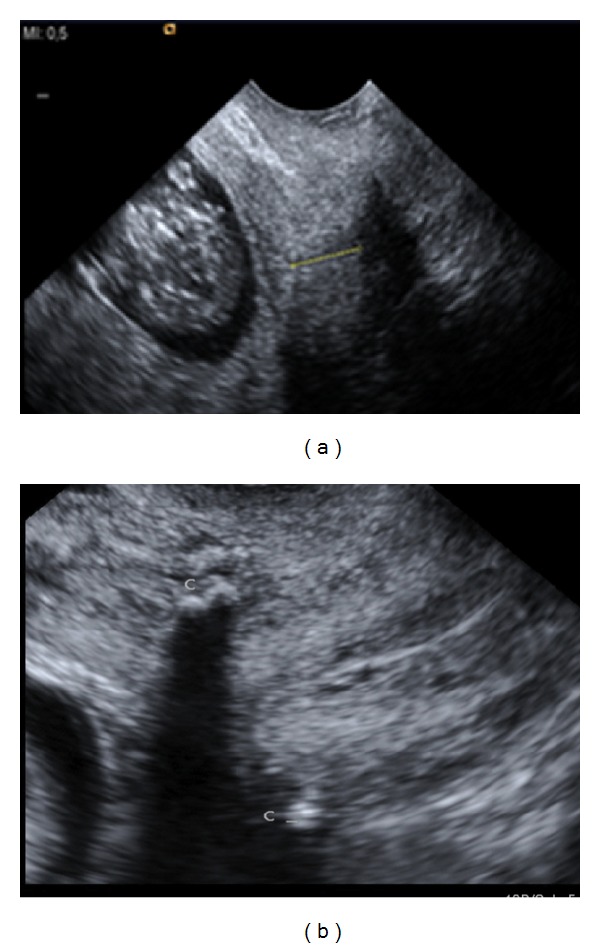
Inner cervical length of 11.4 mm at 12 + 4/7 gestational weeks before abdominal cerclage (a) and at 20 weeks after the transabdominal cervicoisthmic cerclage. The Mersilene band is shown as an echogenic structure at the cervicoisthmic junction (C) and seemed to have increased the cervical length to 25 mm (not shown).

A 30-year-old primigravida with dichorionic/diamniotic (DC/DA) twins was referred to our unit of Marburg University at 11 weeks of gestation. Three years before, the patient had been exposed to two radical surgical conizations because of severe cervical dysplasia and carcinoma in situ, which was only completely removed after a second conization. Speculum examination showed a blind vaginal top without cervix. Transvaginal sonography (TVS) at 12 weeks demonstrated a short inner cervical length of 11.4 mm (below the 3rd centile), without funneling (Figure 1(a)).

**Figure 2:**
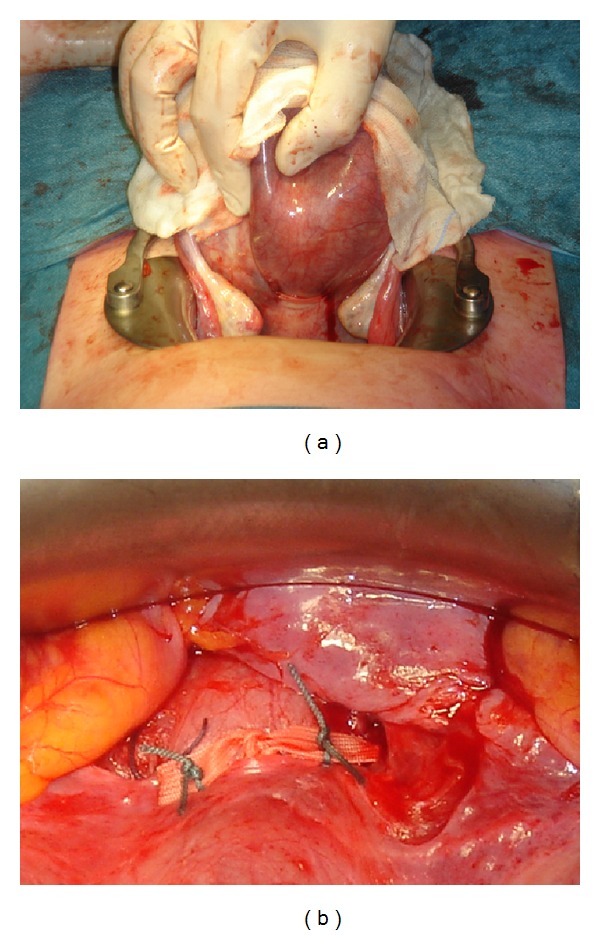
Dorsal (a) and ventral (b) view of the Mersilene band, located at the cervicoisthmic junction. The band is tied on the anterior side of the cervix and the cut ends are fixed to the band with thin nonabsorbable sutures (b).

The patient was counseled for high risk of preterm delivery associated with short cervix and twin gestation, using the publication of Lotgering [4] as a handout. The advantages and disadvantages of expectant management, Arabin pessary, transvaginal cerclage, and transabdominal cerclage were discussed. In an effort to optimize chances to reach viable gestational age we elected to perform transabdominal cerclage. The operation took place at 12 4/7 weeks under general anesthesia at the Radboud University Nijmegen Medical School, Nijmegen, as previously described [2, 3]. In short, after exposing the uterus, the cervicoisthmic junction was visualized and the cerclage (5 mm wide Mersilene ribbon, Ethicon, Norderstedt, Germany) was inserted through the avascular triangle between the ascending and descending branches of the uterine artery on each side (Figures 2(a) and 2(b)). The patient was discharged three days after surgery. TVS showed a cervical length (CL) of 25 mm below the cerclage.

Pregnancy proceeded normally. Regular TVS showed no significant change of CL throughout pregnancy (Figure 1(b)). At 34 3/7 weeks of gestation the patient developed moderately severe preeclampsia. Cesarean delivery was performed, resulting in the birth of a healthy male and female infants with birth weights of 2465 g and 1930 g, respectively. Apgar scores were 7/8/9 and 6/8/8 and umbilical artery pH values were 7.31 and 7.26, respectively. The infants were admitted to the neonatal unit and experienced no complications. The Mersilene band was left in place as suggested by Cammarano et al. [5] for the benefit in any future pregnancy. Lochia flow was normal.

## 3. Discussion

One may question the validity of the decision to perform a cerclage in a woman with no history of preterm birth only because she had an internal 11 mm short cervix while she was carrying twins. On the one hand, the chance of preterm birth before 35 weeks is about 25% in women with singleton pregnancy and a cervical length of 11 mm, in the absence of a history of spontaneous preterm birth (SPTB) [11]. That risk can be reduced to 13% by cerclage [11]. The larger uterine distension in a twin pregnancy may be expected to increase the risk. On the other hand, however, cervical cerclage in twins is controversial to the extent that Berghella and Seibel-Seaman stated that there is no evidence that cerclages should be performed in any woman carrying a multiple gestation [12]. That statement is based on women who received transvaginal cerclage after cervical shortening, not on transabdominal cerclage in women with a cervix too short for transvaginal cerclage. In our patient we performed TAC because our patient and we considered the risk of expectant management too high.

There are different techniques of abdominal cerclages. The classic approach is by TAC during pregnancy, while some authors support the procedure prior to pregnancy [1, 5–10, 12–15]. More recently, laparoscopic transabdominal cervicoisthmic cerclage (LTCC) [10, 14] and even robotic techniques have been described [15]. In addition, one may consider performing TAC in the same session with trachelectomy performed for malignancy [16].

The main advantage of the classical TAC performed during pregnancy is that the cervix has reached its full pregnant size which allows for optimal tension of the cerclage ribbon at knotting and a reported take-home baby rate of 93% [3]. When the cerclage is performed in the nonpregnant state, one has to adjust for the estimated swelling of the cervix in the subsequent pregnancy, which may result in suboptimal tension during pregnancy. We know of women with TAC being too loose resulting in recurrent mid-pregnancy loss and women with TAC being too tight cutting through the cervix as a result of pressure necrosis. In the largest cohort study up to now, Lotgering et al. [3] reported a take-home baby rate of 93% and a rate of only 7% of SPTB before 32 weeks after TAC in 101 in women with suspected cervical insufficiency and a cervix too short for vaginal cerclage. This series included 7 twin pregnancies (personal communication F. Lotgering).

Data on TAC in twin pregnancies are scarce. A systematic review of the literature in PubMed and EMBASE for the match terms “twin pregnancy” and “transabdominal cerclage” revealed 6 publications [5–10] with 8 twin pregnancies. By inclusion of the 7 twin pregnancies from the dataset of Lotgering et al. [3], a total of 15 twin pregnancies with TAC were available for analysis. These cases resulted in 26/30 (87%) healthy infants and 4/30 (13%) perinatal deaths [5, 8] (Table 1). In one woman both twins died from extreme prematurity after SPTB at 20 weeks [8]. TAC was history-indicated in this case, because of failed vaginal cerclage in the previous pregnancy. Two other women delivered a stillborn and a viable infant, one after PPROM at 25 weeks [3] and the other at 37 weeks [8]. Notably, stillbirth is not a common finding in cervical insufficiency or cerclage. We admit that failed cases tend to be hidden for obvious purposes and frequently not published.

From the total group, 14/15 women had TAC by laparotomy, one by laparoscopic transabdominal cervicoisthmic cerclage (LTCC). Three of the 15 interventions took place before the index twin pregnancy, either preconceptionally or in a former pregnancy. The remaining operations took place during the index twin pregnancy and were performed between 12 and 14 gestational weeks. Gestational age at delivery ranged from 20 to 38 weeks: 1/15 at 20 weeks, 3/15 < 32 weeks, 4/15 between 32 and 37 weeks, and 7/15 after 37 weeks. There was no case of delayed interval delivery. Unfortunately, the study population is too small to allow a firm conclusion on a possible difference in outcome of twins compared to singletons after TAC.

Although TAC appears to be an efficient procedure in most women with cervical shortening, one may consider the risks of morbidity by performing two laparotomies. The first is required for the placement of the TAC and the second for the cesarean delivery of the baby. Alternatively, one may consider the possibility of the transvaginal cervicoisthmic cerclage (TCC) using a polypropylene sling as an alternative to the TAC in women presenting with previous cerclage failure. Deffieux et al. [17, 18] reported an overall neonatal survival rate of over 90% in patients with TCC. In this respect, TCC may be a useful alternative for this high-risk patient group.

In the absence of formal proof of efficacy of TAC in twin pregnancies, the decision to perform or not to perform a TAC in a woman at very high risk of immature or preterm delivery because of a severely damaged clinically not existing cervix will remain controversial. The limited data in twin pregnancies suggest that the results of TAC are comparable to those in singleton pregnancies and indications for TAC may be justifiable even in multiple gestations.

## Figures and Tables

**Table 1:** Summary of publications on transabdominal cerclage in twin pregnancies.

Authors (number of twin pairs)	Technique	Gestational week of placement	Pregnancy	Gestational week of delivery	Outcome	Complications
Cammarano et al. [5] (2)	TAC	14 13	3rd 3rd	38 33 4/7	2 alive 2 alive	None

Işçi et al. [6] (1)	TAC	In former pregnancy	2nd	33	2 alive	Polycystic ovary syndrome, congenital hypoplasia, ovary tumor IVF/clomiphene citrate treatment

Lee et al. [7] (1)	TAC	12 5/7	1st	30 5/7	2 alive	History of microinvasive adenocarcinoma of the cervix with VRT Vaginal spotting (29 weeks) contractions, vaginal bleeding (30 weeks)

Olatunbosun et al. [8] (2)	TAC	12–14	?	20 37	2 perinatal deaths 1 stillborn, 1 alive	Preterm labor ?

Olawaiye et al. [9] (1)	TAC	Preconceptional	1st	36	2 alive	Cerclage as part of VRT IVF/ET

Pereira et al. [10] (1)	LTCC	Preconceptional	2nd	38	2 alive	Hysteroscopic metroplasty (uterus subseptus)

Lotgering et al. [3] (7)*	TAC	End of 1st trimester		25 (1) >32 (6)	1 alive; 1 perinatal death 12 alive	PPROM None

*Personal communication, details not presented in the text.

ET: embryo transfer; IVF: in vitro fertilization; LTCC: laparoscopic transabdominal cervicoisthmic cerclage; PPROM: preterm premature rupture of membranes; TAC: transabdominal cervicoisthmic cerclage; VRT: vaginal radical trachelectomy.
